# Titanium-Doped P-Type WO_3_ Thin Films for Liquefied Petroleum Gas Detection

**DOI:** 10.3390/nano10040727

**Published:** 2020-04-11

**Authors:** Yuzhenghan He, Xiaoyan Shi, Kyle Chen, Xiaohong Yang, Jun Chen

**Affiliations:** 1 Key Laboratory of Functional Materials of Chongqing, School of Physics & Information Technology, Chongqing Normal University, Chongqing 400047, China; yuzhenghanhe@cqnu.edu.cn (Y.H.); shixiaoyan@cqnu.edu.cn (X.S.); 2 Department of Bioengineering, University of California, Los Angeles, Los Angeles, CA 90095, USA; kylejchen@g.ucla.edu

**Keywords:** WO_3_, p-type semiconductor, Ti-doped, LPG gas-sensing

## Abstract

Gas sensors are an important part of smart homes in the era of the Internet of Things. In this work, we studied Ti-doped P-type WO_3_ thin films for liquefied petroleum gas (LPG) sensors. Ti-doped tungsten oxide films were deposited on glass substrates by direct current reactive magnetron sputtering from a W-Ti alloy target at room temperature. After annealing at 450 °C in N_2_ ambient for 60 min, p-type Ti-doped WO_3_ was achieved for the first time. The measurement of the room temperature Hall-effect shows that the film has a resistivity of 5.223 × 10^3^ Ωcm, a hole concentration of 9.227 × 10^12^ cm^−3^, and mobility of 1.295 × 10^2^ cm^2^V^−1^s^−1^. X-Ray diffraction (XRD) and X-ray photoelectron spectroscopy (XPS) analyses reveal that the substitution of W^6+^ with Ti^4+^ resulted in p-type conductance. The scanning electron microscope (SEM) images show that the films consist of densely packed nanoparticles. The transmittance of the p-type films is between 72% and 84% in the visible spectra and the optical bandgap is 3.28 eV. The resistance increased when the films were exposed to the reducing gas of liquefied petroleum gas, further confirming the p-type conduction of the films. The p-type films have a quick response and recovery behavior to LPG.

## 1. Introduction

As we continue to progress into the era of the Internet of Things, our lives will undergo significant change. It is predicted that there will be 50 billion sensors connected to the Internet by the year 2020, with various sensor types being the key components of this territory [1,2,3,4,5,6,7,8,9,10,11,12,13,14,15,16,17,18,19,20,21,22,23,24,25,26,27,28,29,30]. Gas sensors are of great use in monitoring hazardous and toxic gases, especially for newly furnished houses, where numerous toxic gases may exist, thus making gas sensors an important component of smart homes [31,32,33,34,35]. Metal oxide semiconductor (MOS)-based gas sensors, whose resistance changes under different degrees of gas concentration, are currently widely used in industrial and domestic applications to detect the concentration and types of gas present. Many MOSs are appropriate for gas detection, such as tungsten trioxide (WO_3_) [36,37,38,39], tin oxide (SnO_2_) [40,41,42], and zinc oxide (ZnO) [43,44]. These gas sensors attract considerable attention because of their high sensitivity, flexibility in production, low cost, and suitability for detecting both reducing and oxidizing gases. However, there are many limitations of MOS-based gas sensors for practical usage. Researchers have focused on improving sensor sensitivity, selectivity, response and recovery time, etc. We know that common MOSs are n-type semiconductors whose resistance may go beyond the detection limits of conventional circuitry when exposed to oxidative gases. P-type gas-sensing metal oxide semiconductors can overcome this limitation while preserving the advantages of these kind of semiconductors [45,46].

Among metal oxide semiconductors, WO_3_ is the most widely applied gas sensing material because it is a chemically and thermally stable semiconductor at room temperature. Moreover, WO_3_ has the advantage of being easily synthesized into nanoparticles and nanofilms via inexpensive and simple methods [47]. Many research groups focus their research on nanostructured n-type WO_3_ based gas sensors for NO*_x_*, H_2_, CO, H_2_S, and NH_3_ as well as some organic gases [36,37,38,39,48,49,50,51]. As previous papers seldom focused on p-type WO_3_ semiconductors, this study aims at preparing p-type WO_3_ thin films via doping. Devices based on p-type semiconductor are indispensable for integration systems. We know that common MOSs strictly in the field of sensors are n-type semiconductors whose resistance may go beyond the detection limits of conventional circuitry when exposed to oxidative gases. P-type gas-sensing metal oxide semiconductors, on the other hand, can overcome this limitation while preserving the advantages of this kind of semiconductor. WO_3_ films are transparent and p-type transparent film can also be used in light-emitting diodes, which is another research hotspot. Liquefied petroleum gas (LPG), as a form of green energy, plays an increasingly important role in our daily lives. More environmentally friendly than gasoline, LPG can replace traditional fuel sources and provide power for automobiles, kitchen appliances, and more. The leakage of LPG in the home is very dangerous, posing a great threat to home safety. In the era of the Internet of Things, the inclusion of highly sensitive LPG sensors in the construction of smart homes is incredibly desirable.

In this study, undoped and Ti-doped WO_3_ thin films were prepared using DC magnetron sputtering. Ti was chosen as the dopant because the radius of Ti^4+^ is similar to that of W^6+^. This may result in the replacement of W^6+^ by Ti^4+^ in film samples. The structure and morphology of the films were studied by XRD and SEM. The electrical, optical, and gas-sensing properties of the p-type films are reported and the origin of the p-type semiconductor is explained. This study indicates that p-type thin film gas sensors can be connected to the Internet of Things and used in a smart house safety system as well as for leakage detection during long-distance transport of LPG.

## 2. Experiment

Pure and Ti-doped tungsten oxide films were prepared on glass substrates by direct current reactive magnetron sputtering at room temperature for 90 min, using a tungsten target (99.95% purity) and an alloy target of W/Ti (97/3.0 wt.%). DC magnetron sputtering is a well-developed method for the preparation of semiconductor thin films. Thin films prepared by such a method are fine and close, evenly distributed, quickly formed, and large in size. Furthermore, we can obtain doped thin films by adding doping elements in any proportion to targets, and the generating procedure is easy to control. The distance between the target and the substrate was approximately 7 cm. The deposition chamber was evacuated to a base pressure of 6 × 10^−4^ Pa. A mixture of nitrogen (99.99%) and oxygen (99.99%) was used as the sputtering gas. The ratio of the partial pressure of oxygen to the constant total pressure (1.3 Pa) was 75% and the oxygen flow rate during sputtering was 100 SCCM (standard cubic centimeters per minute). Under an electric field, the argon ion was accelerated to bombard the W/Ti alloy target. Then, W and Ti atoms and oxygen were deposited on the substrate’s surface by sputtering and grown into film. We can prepare six film specimens simultaneously, with the specimens named TTO. The as-deposited films (marked as TO1 and TTO1) were annealed at 450 °C in N_2_ ambient for 60 min and marked as TO2 and TTO2.

The electrical properties of WO_3_ thin films were determined by a HMS-3000 Hall-effect measurement system (Ecopia Corp., Anyang, South Korea) at room temperature. The crystal structure was analyzed by an X-ray diffractometer (XRD-6000, Shimadzu Corp., Kyoto, Japan). The binding energy and atomic ratio of the films were investigated by ESCALAB 250 X-ray photoelectron spectra (Thermo Fisher Scientific Inc., Waltham, MA, USA) with an incident X-ray energy of 1486.8 eV (Al Ka) and the binding energy (BE) of the C 1s peak (284.8 eV) in the spectrum as the internal reference for energy calibration. The transmission measurements were carried out using a U-4100 spectrophotometer (Hitachi High-Tech America Inc., Schaumburg, IL, USA) in the wavelength range of 300–1000 nm. The gas-sensing characteristics were studied by measuring the electrical resistance of TTO2 (6 mm × 6 mm) in dry air and 10,000 ppm LPG (liquid petroleum gas). The Ohmic contacts of the films and tiny copper wires were established by silver paste. The measurements were performed by placing the film sensors in an airproof quartz glass tube (internal diameter 12.5 mm, length 400 mm) with a gas inlet and an outlet on both ends. The test gas flux rate was set to 100 mL/min. A heater was placed under the tube to control the operating temperature of the film sensors. The resistance and temperature of the sensors were measured by an external Keithley2700 Multimeter/Data Acquisition System (Tektronix Inc., Beaverton, OR, USA). The Keithley 2700 is a multichannel data collector that can test resistance and temperature at the same time, where a thermoelement is used to obtain the temperature signals.

## 3. Results and Discussion

### 3.1. Structure Characterization

From previous studies, we know that MOS gas-sensing materials need a relatively high working temperature. Therefore, except for the XRD, all of the as-prepared samples in this work were annealed at the temperature above the gas-sensors’ working temperature before the sensor was tested with other technologies.

We first investigated the film thickness and microstructure of the samples using a surface profiler and SEM. The thicknesses of the TO2 and TTO2 annealed at 450 °C are 356 and 383 nm, respectively.

**Figure 1:**
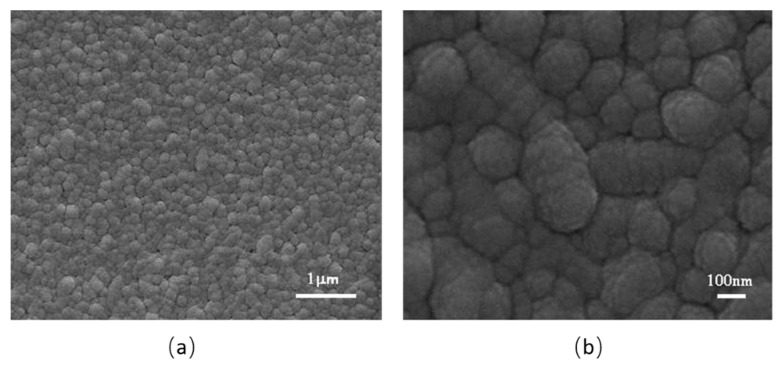
The SEM images of the sample of TTO2: (**a**) is the image for TTO2 films, and (**b**) is for the nanoparticles which form the film.

Figure 1 shows SEM images of TTO2. These images indicate that these films are composed of uniform nanoparticles (Figure 1a). The average diameter of WO_3_ nanoparticles is in the range of 80–120 nm (Figure 1b).

**Figure 2:**
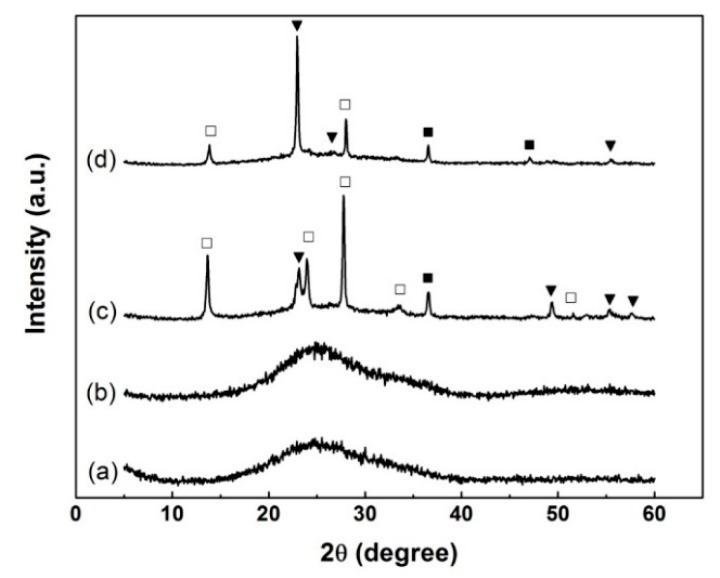
XRD patterns of (**a**) TO1; (**b**) TTO1; (**c**) TO2; (**d**) TTO2: ▼, WO_3_; □, W18O49; ■, W25O73.

The results of the room temperature Hall-effect measurements are shown in Table 1. It can be seen that TO2 is of n-type conductivity and TTO2 is of p-type conductivity. To examine the reliability of the results, the measurements were carried out three times and similar results were observed. XRD patterns of pure and Ti-doped tungsten oxide films before and after annealing at 450 °C for 60 min are shown in Figure 2 (some weak peaks are not tagged). The as-deposited films are amorphous whereas the annealed films are a three-phase mixture of WO_3_ (JCPDS 712141), W18O49 (JCPDS 841516), and W25O73 (JCPDS 710070) crystal. Here, JCPDS stands for Joint Committee on Powder Diffraction Standards. Except for monoclinic tungsten oxide, no extra diffraction peaks from Ti-related phases or impurities were observed.

The lattice parameters for WO_3_ phase were deduced from the XRD data of TO2 and TTO2 and are listed in Table 2. Each lattice parameter (a–c) of TTO2 is larger than the corresponding one of TO2 by about 5.5 pm for WO_3_ phase. This is because W^6+^ is displaced by Ti^4+^ in the lattice of WO_3_ crystal and the radius of Ti^4+^ (68 pm) [52] is larger than that of W^6+^ (62 pm) [53]. Ti becomes an acceptor after substituting into the W site.

**Figure 3:**
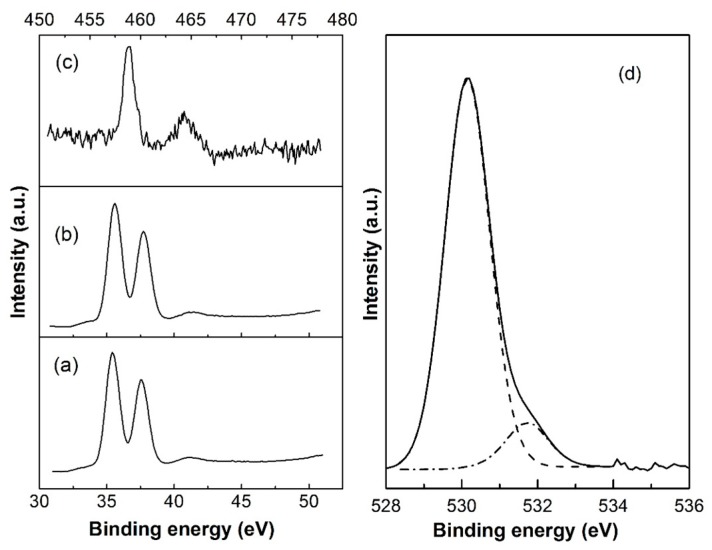
XPS spectra of (**a**) W 4f of TO2, (**b**) W 4f of TTO2, (**c**) Ti 2p of TTO2, and (**d**) O1s-level of TTO2.

In the XRD patterns, we could not find any peaks associated with Ti. The substitutional doping of Ti is a kind of speculation according to the XRD and calculation results. To further confirm the existence of Ti, XPS analysis of TO2 and TTO2 was performed and the results are shown in Figure 3. A well-resolved double peak of XPS for TO2 is shown in Figure 3a, with the highest peak (W 4f_7/2_) at 35.42 eV. This value is close to the reported value of 35.62 eV for W^6+^ in the monoclinic WO_3_ compound [54]. In addition to the stoichiometric WO_3_ peaks, there is also a small shoulder at 33.8 eV that is a clear indicator of the presence of low-valence W of the substoichiometric WO_3_-*_x_* [55,56].

A very similar spectrum is shown in Figure 3b for TTO2. However, the W 4f_7/2_ peak of TTO2 shifted to a greater energy of 35.58 eV, which is due to the substitution of W^6+^ by Ti^4+^. The shoulder at 33.96 eV shows the presence of WO_3−_*_x_* in TTO2 as well. The XPS spectrum of Ti 2p is shown in Figure 3c. The peaks at 458.4 eV and 463.8 eV are ascribed to Ti 2p_3/2_ and Ti 2p_1/2_ of the Ti^4+^, respectively. The values of the peak positions are a little lower than the reported values of 458.95 eV and 464.49 eV for Ti^4+^ in TiO_2_ [57]. Ti incorporation into the WO_3_ lattice is thought to be the reason for the shift of Ti 2p XPS peaks. The atomic ratio of W/Ti is 90.09%:9.91% in TTO2.

Figure 3d shows the results of decomposition into peaks of the O1s-line for the TTO2 samples. The peaks with maxima EpO1s = 530.1 eV correspond to O1s-levels of oxygen atoms O^2−^ in the lattice and the maxima of peaks in region 531.7 eV belong to weakly adsorbed species and O^−^ oxygen states.

### 3.2. Energy Band Structure of the TTO and TTO2

**Figure 4:**
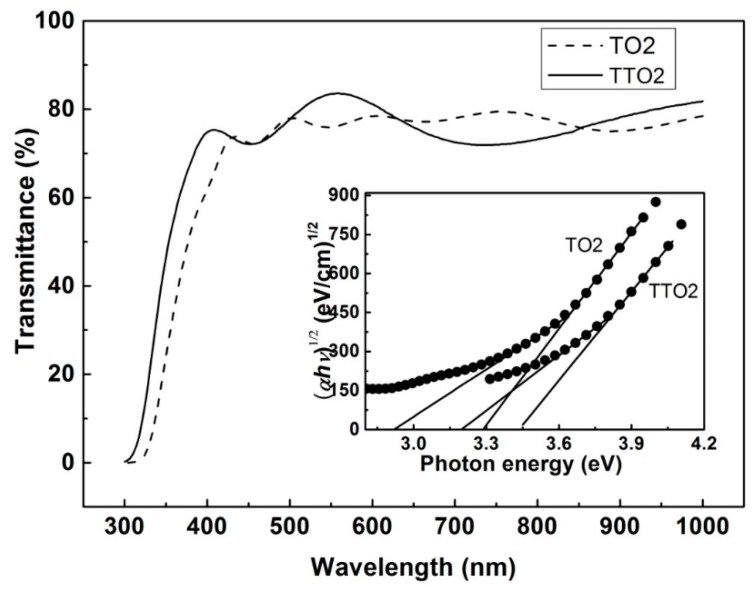
Optical transmittance spectra of TO2 and TTO2. The inset shows the plots of (*αhν*)^1/2^ against *hν*.

In order to compare the optical bandgap of TTO2 and TO2, the transmittance spectra of both films were measured, as shown in Figure 4. The transmittance of TTO2 is between 72% and 84% in the visible spectra. The method developed by *T*auc was used to determine the optical bandgap. The absorption coefficient α is given by [49]

*α* = *A*(*hν* − *E_g_*)*^r^*/*hν* (1)

where *A*, *hν*, and *E*_g_ are constants of proportionality, photon energy, and optical bandgap energy, respectively. TTO2 has a direct bandgap for *r* = 1/2 and 3/2 and an indirect bandgap for *r* = 2 and 3. From the inset in Figure 4, the indirect optical bandgaps of TO2 and TTO2 are estimated to be 3.06 and 3.28 eV, respectively. The optical bandgap of TO2 agrees with the reported value [58].

### 3.3. Gas Sensing Performances

We used dry air as the reference gas in the gas-sensing test. The tiny copper wires coming out of the film sensor and the thermoelement were connected to the data acquisition card of the 2700 Multimeter/Data Acquisition System. To begin, we injected dry air into the glass tube for 10 min to exhaust the unknown gas in the tube. Next, we heated the film sensor to its operating temperature. After the sensor reached a constant value of resistance at its operating temperature, we stopped injecting dry air and filled the tube with 10,000 ppm LPG at a certain flow rate. After the sensor reached a new constant and steady resistance level, we stopped injecting 10,000 ppm LPG gas, filled the tube with dry air again, and waited for the sensor to recover its previous resistance level. This is the process of one experiment procedure. Then, we increased the temperature and repeated the previous steps for the next test cycle. The data of the experimental temperature and the sensor’s resistance value during the entire process were recorded by the Keithley 2700 Multimeter/Data Acquisition System and stored in the computer automatically.

**Figure 5:**
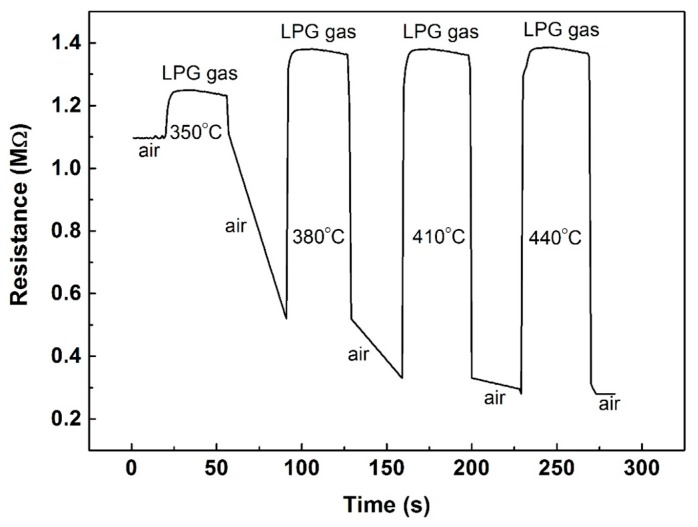
The response and recovery behavior of TTO2 to 10,000 ppm liquefied petroleum gas (LPG) at different temperatures.

The response and recovery characteristics of TTO2 to 10,000 ppm LPG in dry air at different temperatures are shown in Figure 5. Figure 5 shows four measurement cycles within one test working continuously at different temperatures. LPG is a mixture of hydrocarbons similar to n-propane and n-butane, indicating that LPG is a type of reducing gas, which can offer an electron. The reaction of the film surface will lead to a lower surface coverage of adsorbed oxygen when n-type tungsten oxide films are exposed to LPG-containing air—this property results in a decrease in the resistance of the film [59]. However, the resistance of TTO2 increases when the films are exposed to LPG, as shown in Figure 5.

**Figure 6:**
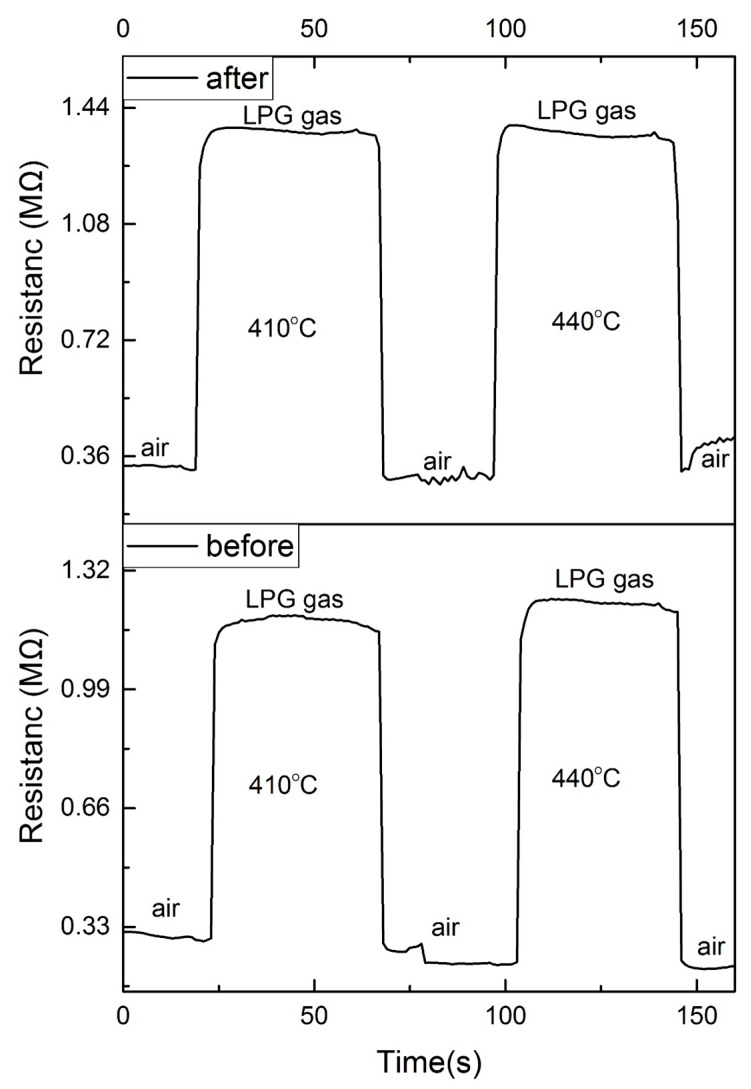
The response and recovery behavior of TTO2 to 10,000 ppm LPG before and after one week.

To further confirm the performance of TTO2, we repeated the test twice within a week and the results are shown in Figure 6. We can see a similar behavior of TTO2 from Figure 6: the resistance of TTO2 increases when the films are exposed to LPG after one week. This resistance change regularity of TTO2 to reducing gases further demonstrates that TTO2 is a p-type semiconductor because the combination of the injected electrons with free holes will reduce the concentration of the majority of carriers for a p-type semiconductor.

The gas sensitivity is defined as the ratio of the resistance in the reducing atmosphere to the resistance in air. The response (or recovery) time is defined as the time required for the sensor when its resistance changes to 90% of the maximum value (or minimum value) in one test cycle. The sensitivities to LPG for TTO2 are 1.14 at 350 °C, 2.65 at 380 °C, 4.18 at 410 °C, and 4.93 at 440 °C. The response and recovery time are within 10 s in the temperature range 350–440 °C. The recovery time is shorter than the response time, so we can see that there is a smooth angle on the rising edge and an approximate right angle on the falling edge of the curve. In this work, the minimum working temperature was 350 °C. Lowering the operating temperature will be our next step.

We now look into the corresponding mechanism. The holes on the impurity energy level jump down to the valence band as the operating temperature rises, resulting in the decrease of the resistance of TTO2. When LPG gas (whose main chemical components are C_3_H_8_ and C_4_H_10_) is injected into a glass tube, the reactions on the surface of TTO2 can be expressed by the following equations:

$$C_{3}H_{8}+{10O}_{\mathrm{abs}}^{-}\to {4H}_{2}O+{3\mathrm{CO}}_{2}+10e$$

$$C_{3}H_{8}+{10O}_{\mathrm{abs}}^{2-}\to {4H}_{2}O+{3\mathrm{CO}}_{2}+20e$$

$$C_{4}H_{10}+{13O}_{\mathrm{abs}}^{-}\to {5H}_{2}O+{4\mathrm{CO}}_{2}+13e$$

$$C_{4}H_{10}+{13O}_{\mathrm{abs}}^{2-}\to {5H}_{2}O+{4\mathrm{CO}}_{2}+26e$$

where $O_{\mathrm{abs}}^{-}$ and $O_{\mathrm{abs}}^{2-}$ represent the oxygen ions adsorbed on the surface of TTO2, and e represents an electron. The electrons released during the reaction process will combine with the holes in the valence band, and the resistance will increase.

## 4. Conclusions

P-type transparent tungsten oxide films were fabricated by Ti doping. The p-type tungsten oxide films are a monoclinic system, with a hole concentration of 9.227 × 10^12^ cm^−3^, a mobility of 1.295 × 10^2^ cm^2^V^−1^s^−1^, and a resistivity of 5.222 × 10^3^ Ωcm at room temperature. The gas-sensing properties show that Ti-doped WO_3_ films are p-type semiconductors and have a quick response and recovery behavior to reducing gases. The changes in the lattice parameters and the W 4f and Ti 2p binding energies reveal that p-type conductance originates from the substitution of W^6+^ by Ti^4+^. The optical bandgap of the p-type films was found to be 3.28 eV and the transmittance was found to be between 72% and 84%. Further experiments are in progress to increase the hole concentration and gas sensitivity of p-type tungsten oxide films, which can be integrated with vibration-mode nanogenerators to fabricate self-powered gas sensors to detect the leakage of LPG in long-distance transport.

## Figures and Tables

**Table 1:** Room temperature electrical properties of TO2 and TTO2.

Sample	Type	Carrier Density (cm^−3^)	Resistivity (Ωcm)	Mobility (cm^2^V^−1^s^−1^)
TO2	n	3.729 × 10^11^	1.579 × 10^4^	1.060 × 10^3^
TTO2	p	9.227 × 10^12^	5.223 × 10^3^	1.295 × 10^2^

**Table 2:** Lattice parameters of TO2 and TTO2 for WO_3_ phase.

Sample	Phase	a (Å)	b (Å)	c (Å)
TO2	WO_3_	7.300	7.542	7.610
TTO2	WO_3_	7.351	7.599	7.656
